# CREATION OF EVIDENCE-BASED GERIATRIC SURGERY STANDARDS

**DOI:** 10.1093/geroni/igz038.1485

**Published:** 2019-11-08

**Authors:** Marcia McGory Russell

**Affiliations:** UCLA Medical Center, Los Angeles, California, United States

## Abstract

The aim of this abstract is to describe how to establish high-quality, valid standards to improve surgical care of the older adult. The older adult population has high demand for high-quality surgical care. Building upon prior guidelines, quality indicators, and pilot projects, the Coalition for Quality in Geriatric Surgery (CQGS) included 58 diverse stakeholder organizations committed to improving surgery for older adults. Using a modified RAND-UCLA Appropriateness Methodology, 44 of 58 CQGS Stakeholders twice rated validity (primary outcome) and feasibility for 308 standards, ranging from goals and decision-making, pre-operative assessment and optimization, perioperative and postoperative care, to transitions of care beyond the acute care hospital. Stakeholders rated the vast majority of standards of care as highly valid (99%) and feasible (94%) for improving the quality of surgical care provided to older adults.

